# Collapsing Perisomatic Inhibition Leads to Epileptic Fast-Ripple Oscillations Caused by Pseudosynchronous Firing of CA3 Pyramidal Neurons

**DOI:** 10.1523/JNEUROSCI.0500-25.2025

**Published:** 2025-10-02

**Authors:** Dániel Schlingloff, Tamás F. Freund, Balázs Hangya, Attila Gulyás

**Affiliations:** ^1^Laboratory of Cerebral Cortex Research, HUN-REN Institute of Experimental Medicine, Budapest H-1083, Hungary; ^2^MTA–HUN-REN Lendület “Momentum” Laboratory of Systems Neuroscience, HUN-REN Institute of Experimental Medicine, Budapest H-1083, Hungary; ^3^HUN-REN Institute of Experimental Medicine, Budapest H-1083, Hungary

**Keywords:** epilepsy, hippocampus, ripple oscillation, sharp wave, synchrony

## Abstract

Diverse network oscillations, thought to represent different information processing modes of cortical networks, are accompanied by synchronous neuronal activity at various temporal scales. Sharp wave-associated ripple oscillations, supporting memory consolidation in the hippocampus, are among the fastest physiological oscillations characterized by strong interneuronal synchrony. In contrast, when hippocampal activity turns epileptic, pathological fast-ripple oscillations appear. The distinction of the two oscillations is diagnostically relevant; however, how differential mechanisms of the same network generate the two activities is not well understood. Here we addressed this question using an in vitro hippocampal model that allowed targeted recording of cell types and local pharmacological manipulations in mice of either sex. We showed that inhibition did not contribute to current and rhythm generation of fast-ripples, unlike physiological ripple oscillations. Instead, pathological fast-ripples emerged when perisomatic inhibition from parvalbumin-expressing basket cells collapsed and depended on the quasi-simultaneous onset of stereotypical pyramidal cell (PC) bursts leading to pseudosynchrony. This was accompanied by a loss of spatial coherence. In epileptogenic conditions, deep CA3 PCs selectively ramped up their burst activity before fast-ripple onset, while normally nonbursting superficial PCs acquired burst capability. These results point to PC pseudosynchrony as the underlying mechanism of fast-ripples, with differential contribution of known PC types.

## Significance Statement

Sharp wave-ripple oscillations in the hippocampus support memory consolidation via coordinated inhibition-driven synchrony, whereas pathological fast-ripples mark epileptogenic activity. Using an in vitro hippocampal model in mice, we show that fast-ripples emerge from pseudosynchronous bursting of pyramidal cells after perisomatic inhibition collapses. Deep pyramidal cells of the CA3 area of hippocampus ramp up bursting activity before fast-ripple onset, while normally nonbursting superficial cells fire bursts under epileptic conditions. In contrast to ripple oscillations, fast-ripples lack rhythmic inhibition and exhibit degraded spatial coherence. These findings reveal cell type-specific excitability changes and implicate local failure of inhibition and loss of coherence as mechanisms driving fast-ripple emergence.

## Introduction

The hippocampus participates in memory formation with a unique role in reactivation and consolidation of episodic memories (Buzsáki, 1989). It was proposed that this is achieved by the oscillatory multiplexing of different hippocampal population codes that enable flexible reconfiguration of effective network connectivity (Akam and Kullmann, 2014). Specifically, memory consolidation was associated with sharp wave-ripple (SWR) oscillation complexes, which are characterized by what is considered the highest degree of network synchrony in the mammalian brain (Buzsáki et al., 1983).

Sharp waves predominantly occur during consummatory behaviors and slow-wave sleep, when they feature time-compressed “off-line” reactivation of neuronal ensembles (e.g., memory traces) linked together by recent experience during exploratory behavior (Nádasdy et al., 1999; Foster and Wilson, 2006). Self-organized sharp waves arise in the recurrently connected CA3 excitatory network (Buzsáki and Chrobak, 1995; but see also Oliva et al., 2016). Superimposed high-frequency (∼200 Hz) ripple oscillations (RIP) are thought to segment neuronal activity into short bouts with a timing optimized to facilitate long-term synaptic changes in neocortical target areas (McNaughton et al., 1978; Buzsáki, 2015). Accordingly, neuronal interaction between the hippocampus and neocortex coordinated at ripple frequency is thought to be important for the development and retrieval of lasting memory traces (Jadhav et al., 2016; Khodagholy et al., 2017; Vaz et al., 2019).

The strong recurrent connectivity of the hippocampus makes it prone to pathological hypersynchrony resulting in epileptic seizures in temporal lobe epilepsies. In such epileptic states, the hypersynchronous hippocampal activity is associated with pathological high-frequency oscillations commonly referred to as fast-ripples (fast-RIPs). Fast-RIPs are characterized by on average higher, albeit partially overlapping spectral components compared with physiological RIPs (Engel et al., 2009) and known to disrupt normal hippocampal function (Ewell et al., 2019). Fast-RIP occurrence is an emerging biomarker for identifying hippocampal and neocortical epileptogenic areas (Kramer et al., 2019; Birk et al., 2021; Tamilia et al., 2021; Santana-Gomez et al., 2022).

Physiological SWRs are initiated stochastically in the recurrent network of CA3 (Buzsáki and Chrobak, 1995) pyramidal cells (PCs), possibly primed by recently discovered CA3 athorny pyramidal cells capable of complex spike bursting (CSB; Hunt et al., 2018). Subsequent studies identified these cells as deep PCs, noting that while both deep and superficial PCs possess thorny excrescences, they differ in their number and spatial distribution. Accordingly, we adopted this modern terminology. The buildup of excitation recruits a network of synaptically interconnected parvalbumin-expressing basket cells (PVBCs), which convey synchronized, rhythmic inhibition onto PCs, being thus responsible for both current and rhythm generation during physiological RIPs (Schlingloff et al., 2014; Stark et al., 2014; Gan et al., 2017; Valero et al., 2017).

A similar stochastic buildup of excitatory synaptic potentials was observed before epileptiform burst onset as well (de la Prida et al., 2006), while fast-RIPs are known to predominantly represent burst firing of pyramidal cells (Draguhn et al., 1998; Foffani et al., 2007; Ibarz et al., 2010; Bragin et al., 2011). However, the contribution of inhibitory mechanisms to fast-RIP rhythm- and current-generation is unclear. Although depolarization block of PVBCs was proposed to be involved in the generation (Karlocai et al., 2014) and spread of epileptic activity (Trevelyan et al., 2006), the temporal relationship of the onset of PC bursting, PVBC depolarization block, and emergence of fast-RIPs remained unknown (Jiruska et al., 2017).

A better understanding of the underlying mechanisms of fast-RIP generation is important to help distinguish them from physiological RIPs, which is a clinically relevant task not yet fully resolved (Engel et al., 2009; Liu et al., 2022). To address this, we used an in vitro hippocampal model capable of spontaneous SWR generation under physiological conditions, as well as interictal epileptiform discharges (IEDs), consistently accompanied by fast-RIPs, induced by elevated extracellular potassium. In this preparation, fast-RIPs emerged as a temporally precise component of IEDs, providing a platform to study the cellular and network mechanisms underlying fast-RIP generation. Using local pharmacological manipulations and targeted single-cell recordings, we dissected the contribution of PVBCs and known PC subtypes to fast-RIP generation.

## Materials and Methods

### Animals

Wild-type (C57BL/6J, *n* = 29) mice of both sexes (*n* = 19/29 males, postnatal days ∼30) were used. To record selectively from cells containing the Ca^2+^ binding protein parvalbumin, transgenic mice (*n* = 5, *n* = 4/5 males) expressing enhanced green fluorescent protein (eGFP) controlled by the parvalbumin promoter were also used in this study (Meyer et al., 2002). Mice were kept in the vivarium on a 12 h light/dark cycle and provided with food and water ad libitum. The animals were housed two or three per cage. All experiments were approved by the Ethical Committee for Animal Research at the Institute of Experimental Medicine, Hungarian Academy of Sciences, and conformed to Hungarian (1998/XXVIII Law on Animal Welfare) and European Communities Council Directive recommendations for the care and use of laboratory animals (2010/63/EU).

### Slice preparation and recording solutions

Mice were decapitated under deep isoflurane anesthesia. The brain was removed and placed into an ice-cold cutting solution, which had been bubbled with 95% O_2_–5% CO_2_ (carbogen gas) for at least 30 min before use. The cutting solution contained the following (in mM): 205 sucrose, 2.5 KCl, 26 NaHCO_3_, 0.5 CaCl_2_, 5 MgCl_2_, 1.25 NaH_2_PO_4_, 10 glucose, saturated with 95% O_2_–5% CO_2_. Horizontal hippocampal slices of 450 µm thickness were cut using a vibratome (Leica VT1000S). After acute slice preparation, slices were placed into an interface-type holding chamber for recovery. This chamber contained standard ACSF at 35°C that gradually cooled down to room temperature. The ACSF solution contained the following (in mM): 126 NaCl, 2.5 KCl, 26 NaHCO_3_, 2 CaCl_2_, 2 MgCl_2_, 1.25 NaH_2_PO_4_, 10 glucose, saturated with 95% O_2_–5% CO_2_ (carbogen). To evoke epileptic activity in slices, normal ACSF was changed to a modified ACSF with the following composition (in mM): 126 NaCl, 3.5 KCl, 26 NaHCO_3_, 1.6 CaCl_2_, 1.2 MgCl_2_, 1.25 NaH_2_PO_4_, and 10 glucose, saturated with carbogen. After 10–15 min, extracellular potassium concentration was raised to 8.5 mM to transform spontaneous SWRs to pharmacologically elicited, spontaneously recurring IEDs. In this preparation, fast-RIPs emerged as a temporally precise component of IEDs and were not observed independently.

### LFP and loose-patch recordings

Recordings were performed under visual guidance using differential interference contrast (DIC) microscopy (Nikon FN-1) and a 40× water immersion objective. After incubation for at least 2 h, slices were transferred individually into a submerged-type recording chamber equipped with a dual superfusion system for improved metabolic supply to the slices (Hájos et al., 2009). In this design, the slices were placed on a metal mesh, and two separate fluid inlets allowed ACSF to flow both above and below the slices at a rate of 3–3.5 ml/min for each flow channel at 30–32°C (Supertech Instruments). Standard patch pipettes filled with ACSF were used for local field potential (LFP) recordings. Patch pipettes were pulled from borosilicate capillaries (with inner filament, thin walled, OD 1.5, Hilgenberg) with a PC-10 puller (Narishige). In all experiments, electrodes were placed in the hippocampal pyramidal layer of the CA3 region for LFP registration. ACSF-containing pipette resistances were 3–6 MΩ. Similar electrodes were used to perform loose-patch experiments. To verify cell identity, loose-patch recorded cells were filled with biocytin [intracellular composition in mM: 110 K-gluconate, 4 NaCl, 20 HEPES, 0.1 EGTA, 10 phosphocreatine, 2 ATP, 0.3 GTP, 3 mg/ml biocytin adjusted to pH 7.3–7.35 using KOH (285–295 mOsm/L)]. After biocytin filling, the slices were fixed in 4% PFA in 0.1 M phosphate buffer (PB; pH 7.4) for ∼24 h, followed by washout with PB several times. Then, sections were blocked with normal goat serum (NGS; 10%) diluted in Tris-buffered saline, pH 7.4, followed by incubations in Alexa Fluor 488- or Alexa Fluor 594-conjugated streptavidin (1:1,000; Invitrogen). Sections were then mounted on slides in Vectashield (Vector Laboratories), and neurons were morphologically identified based on their location and dendritic and axonal arborization. Recordings were performed with MultiClamp 700A or 700B amplifier (Molecular Devices). Data were digitized at 10 or 20 kHz with a DAQ board (National Instruments, USB-6353) and recorded with software developed in C#.NET and VB.NET in the laboratory.

### Intracellular recordings

To investigate potential anatomical and functional differences underlying distinct firing patterns of pyramidal cells recorded in loose-patch configuration, cells were repatched with intracellular solution (with composition stated above). Before whole-cell configuration, cells were allowed to recover their firing pattern in cell-attached configuration. This was used in addition to visual guidance as a second confirmation to verify cell identity (type I/II firing) due to dense packing of the pyramidal cell layer. In whole-cell recordings, PC membrane potential was held at approximately −60 mV, and current injection to test firing pattern was set just above the rheobase (minimum current injection required for suprathreshold response) during the experiment. Recorded cells were visualized as described above, and images were acquired using an FV1000 confocal microscope (Olympus) with either a 20× or a 60× oil-immersion objective. Cells with complete dendritic arbor were reconstructed and analyzed using Neurolucida (MBF Bioscience). For Sholl analysis 10 µm radius increments from the soma were used.

### Local drug administration

To prevent alteration of global network state while pharmacologically dissecting components of LFP generation, local drug application was used in small volumes as described previously (Schlingloff et al., 2014). Briefly, drugs were diluted in a “puffing” solution and injected locally to a small volume of tissue where one LFP recording electrode was placed. Another control electrode was used to verify unaltered global network activity. Riluzole, TTX, biocytin, ATP, GTP, EGTA, and phosphocreatine were purchased from Sigma-Aldrich; gabazine was purchased from Hello Bio. All other salts and chemicals were purchased from Molar Chemicals.

### Digital signal processing and analysis

**Figure 1.**
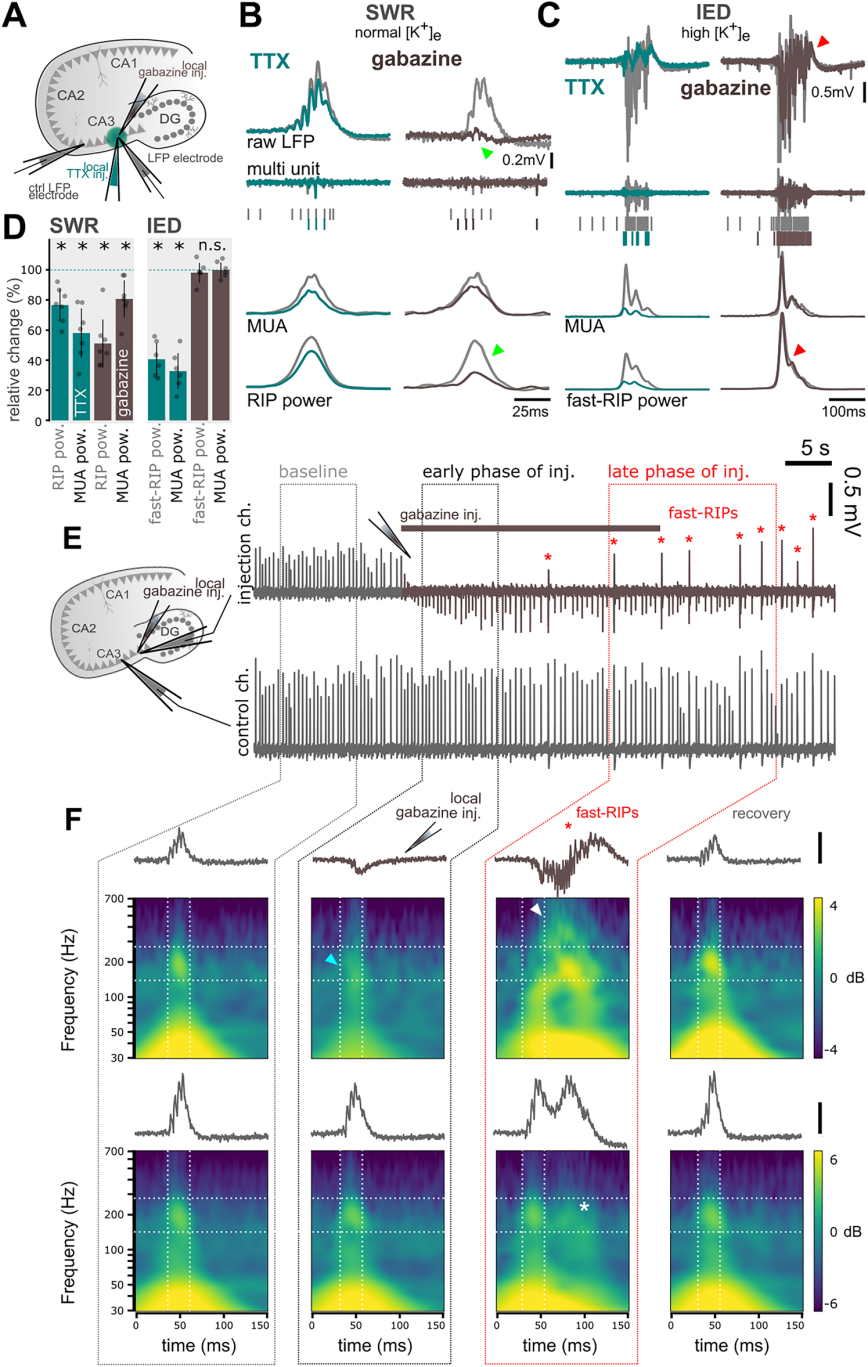
Inhibitory synaptic currents contribute to RIP but not fast-RIP LFP. ***A***, LFP was recorded with a “local” and a “distant” (∼150 µm) electrode. TTX (green) or gabazine (brown) were puffed into the vicinity of the local electrode to focally inactivate AP generation or inhibitory transmission, respectively. ***B***, Top, Average sharp wave-ripple LFP during baseline (gray) and after TTX (green) or gabazine application (brown). Middle, Examples of MUA traces before (gray) and after (green, brown) drug application. Bottom, Average MUA and RIP (150–250 Hz band) power. TTX application only caused a small decrease in MUA and RIP power during SWR, whereas blocking perisomatic GABA_A_ receptors with gabazine caused a large decrease of LFP amplitude and RIP power without affecting MUA power. ***C***, The same as panel ***B*** but during epileptiform activity. During epileptic bursts, TTX significantly decreased the LFP amplitude, MUA power, and fast-RIP (250–500 Hz band) power, whereas gabazine application did not change the LFP, indicating that inhibition does not contribute to fast-RIP LFP. ***D***, Relative change of RIP, fast-RIP, and MUA power compared with baseline (100%, dashed line). TTX (*n* = 7) and gabazine (*n* = 7) both decreased SWR RIP and MUA power (mean ± SD, TTX RIP, −23.4 ± 11%, *p* = 0.02; TTX MUA, −41.9 ± 17%, *p* = 0.01; gabazine RIP, −48.9 ± 17%, *p* = 0.02; gabazine MUA, −19.3 ± 13%, *p* = 0.02, Wilcoxon signed-rank test). TTX significantly decreased both fast-RIP and MUA power during IEDs (*n* = 6), while gabazine (*n* = 6) did not cause any changes (TTX fast-RIP, −59.3 ± 11%, *p* = 0.03; TTX MUA, −67.1 ± 12%, *p* = 0.03; gabazine fast-RIP, −1.85 ± 7%, *p* = 0.24; gabazine MUA, −0.1 ± 5%, *p* = 0.91, Wilcoxon signed-rank test). ***E***, Top, Extended local gabazine application (∼30 s) induced a local epileptogenic area and altered the shape of RIP oscillations (red asterisks), indicating a shift in the generating mechanism. Bottom, SWRs recorded on the distant (control) electrode remain unchanged. ***F***, Example zoomed-in LFP traces from the local (top) and distant (bottom) electrodes, with wavelet power spectra averaged over 50 consecutive RIP or fast-RIP events from four phases of the gabazine injection experiment. SWR and RIP events during baseline (first column) and in the early phase (second column) reproduced the results of the gabazine puff experiment shown in panel ***B***, with a spectral peak around 200 Hz that was strongly suppressed by gabazine (blue arrowhead). In the late phase of gabazine injection (third column), IEDs evolved in the puffed area, characterized by fast-RIP oscillations (white arrowhead). On the distant electrode (bottom), a normal SWR appeared synchronously with the IED, followed by a second SWR-like event (smaller 200 Hz component, white asterisk), likely induced by the epileptiform activity. The activity reverted to the baseline (recovery, fourth column) as perisomatic inhibition recovered (recording from 10 min after the cessation of the 30-seconds-long gabazine injection).

All data were processed and analyzed off-line using self-developed programs written in Delphi 6.0 by A.G. and Matlab 8.5.0 and Python 2.7.0 by D.S. Signals were filtered with a two-way RC filter to preserve phase. All automatic detection steps were supervised. SWR peak times were detected in 30 Hz low-pass filtered field recordings using a threshold value of five times the SD of the signal. We note that ∼200 Hz frequency oscillations in acute hippocampal slices might be coined as ripple-like, to distinguish from ripple oscillations recorded in vivo; however, based on the shared mechanistic properties and consistent with previous literature (Maier et al., 2003, 2012; Behrens et al., 2005; Hájos et al., 2009; Schlingloff et al., 2014; Evangelista et al., 2020), we referred to these events as ripples. We detected the time of negative ripple peaks in an LFP trace bandpass filtered in the ripple frequency band (150–250 Hz), identified the ripple cycle closest to the SWR peak, and used its negative peak as triggering event for averages (Fig. 1*B*) to preserve ripple phase. Ripple power was calculated by low-pass filtering the absolute value of the ripple-band filtered signal. Multiunit activity (MUA) was detected in high-pass filtered (500 Hz) LFP traces. MUA power was calculated by low-pass filtering the absolute value of the MUA-filtered signal. Area under the average ripple and MUA power, triggered by the SWR peak, was compared between baseline and drug application periods. For IEDs, fast-ripple and MUA power was calculated similarly using the bandpass (200–500) or high-pass filtered (500 Hz) LFP signal. The IED peak was defined as the peak of the fast-RIP power, as IEDs and fast-ripples invariably co-occurred in our preparations. Wavelet spectra were calculated from unfiltered data using Morlet wavelet (Torrence and Compo, 1998). To assess ripple phase relationship across recording locations, we performed Wavelet Transform Coherence (WTC) analysis [MATLAB (MathWorks) package from Grinsted et al. (2004)]. WTC was applied to the two unfiltered LFP time series to generate 2-D coherence maps of ripples and fast-RIPs. Statistical comparison of coherence between RIPs and fast-RIPs was performed by comparing average coherence values during sharp waves and IEDs in the respective frequency bands for the events (±25 and ±50 ms around SWR and IED peaks, respectively, 150–250 and 250–500 Hz for SWRs and IEDs, respectively). For quantification of intrinsic and spike shape features of deep and superficial PCs, including subthreshold membrane properties, properties of isolated spikes, and features during current injection protocols, we used the eFEL library (Ranjan et al., 2024). The current injection protocol consisted of a series of hyperpolarizing and depolarizing square current pulses of 800 ms duration and amplitudes between −100 and +100 pA at 10 pA step intervals, then up to 300 pA at 50 pA step intervals and, finally, up to 600 pA at 100 pA step intervals.

**Figure 2.**
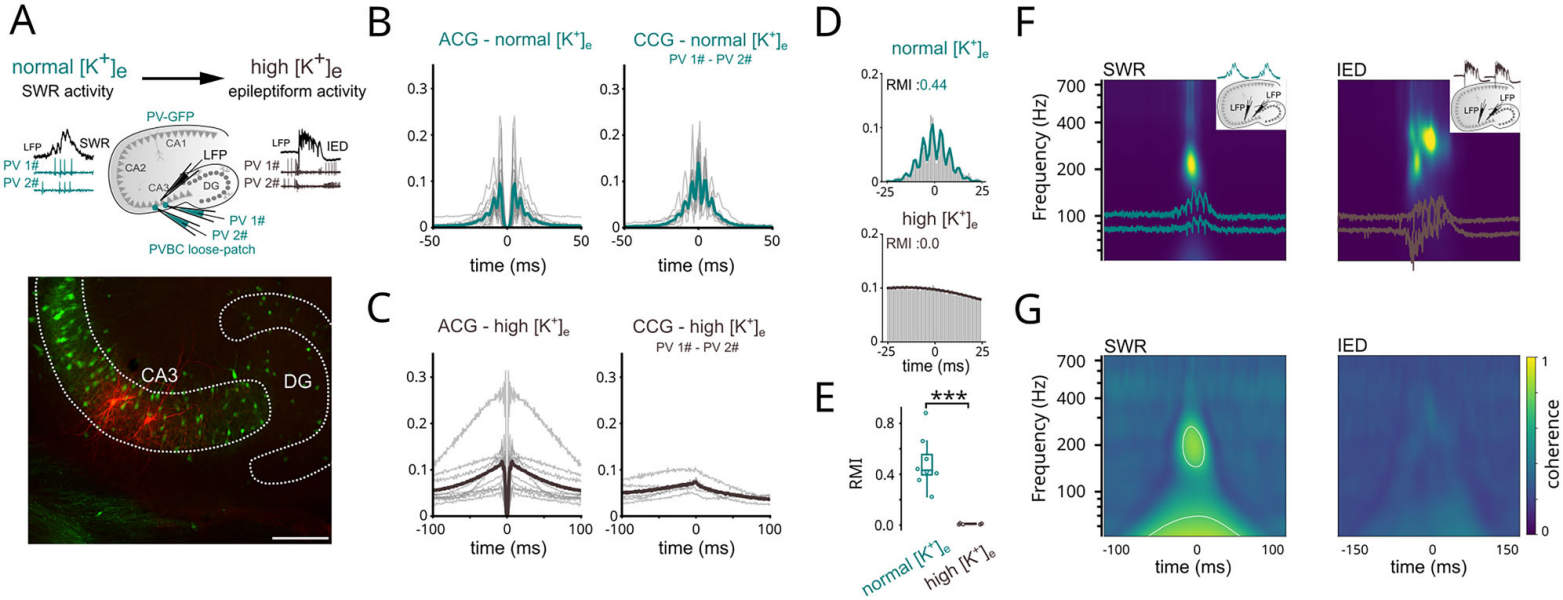
PVBCs are desynchronized during epileptiform activity. ***A***, Top left, Schematic of the experimental configuration (top left). Top right, The spiking of two PVBCs was recorded in parallel with the LFP during sharp waves (top) and subsequently evoked epileptiform activity (IED, bottom) from a PV-GFP mouse. Bottom, Representative image of two recorded and post hoc verified PVBCs (red; scale bar, 200 µm). ***B***, Left, Spike autocorrelograms (ACG) of PVBCs (*n* = 16) recorded during SWR indicated that PVBCs were firing around RIP frequency. Right, spike cross-correlograms (CCG) of PVBC pairs (*n* = 8; distance, 100–300 µm) during SWRs indicated that the cells were synchronized at RIP frequency, and thus generated coherent, oscillating inhibition. ***C***, The same as in panel ***B***, but during epileptiform activity. The same PVBCs lost their rhythmic firing (ACG, left; *n* = 10 cells) and their synchrony (CCG, right; *n* = 5 pairs). ***D***, Ripple modulation index (RMI) of cell pair firing was calculated by fitting a sine wave modulated by a Gaussian window to the individual CCGs. Cofiring at ripple frequency (top) disappeared during epileptiform activity (bottom) in an example pair of PVBCs. ***E***, Population data showing high ripple modulation indices during SWRs (*n* = 8) but not during IEDs (*n* = 5; ripple modulation index during SWRs, mean ± SE 0.48 ± 0.071; during IEDs, 0.01 ± 0.002, *p* < 0.01, Wilcoxon rank-sum test). ***F***, Left, LFP traces of SWRs from two electrodes (200–300 µm apart) are superimposed on the cross-wavelet power spectrum of the recordings (averaged over *n* = 50 consecutive SWRs from 5 slices each, 250 in total), showing a prominent RIP peak at ∼200 Hz. Inset, schematic of the experiment. Right, LFP traces and average cross-wavelet spectrum showed that IED-associated fast-RIP oscillations appeared on both electrodes and shared spectral components above 200 Hz. ***G***, Left, Wavelet coherence of SWRs showed high coherence around RIP frequency. White contour indicates 5% significant level against red noise (χ^2^ test). Right, The coherence plot during IEDs indicated an absence of significant coherence, in contrast to what was seen during SWRs (average coherence during RIPs, median ± SEM, 0.67 ± 0.01; during fast-RIPs, 0.30 ± 0.01; *p* < 0.0001, Wilcoxon rank-sum test).

Spike detection in loose-patch recordings was performed on LFP traces high-pass filtered above 500 Hz using a positive threshold value of three times the SD of the signal. Auto- and cross-correlations were calculated at 1 ms resolution. Auto- and cross-correlations were computed using all spikes recorded during defined network states without applying any event-triggered window, allowing the assessment of overall spike timing relationships across conditions. Zero-lag values were removed from autocorrelations presented in Figure 2*B* and *C* for better visibility.

**Figure 3.**
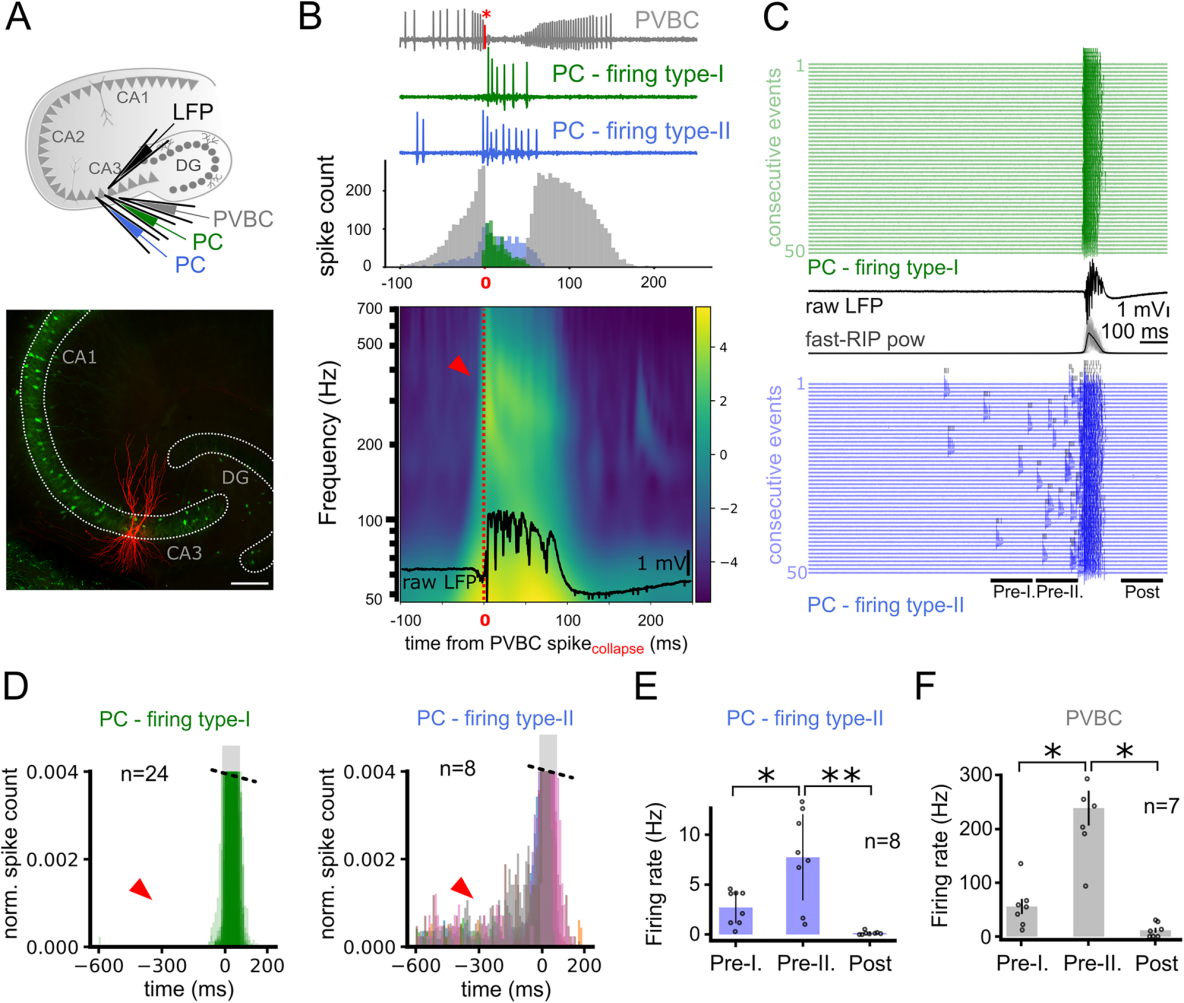
Superficial and deep CA3 pyramidal cells show distinct firing patterns during fast-RIPs. ***A***, Top, Schematic of the recording configuration. LFP recording was combined with loose-patch recording of PVBCs and one or more PCs recorded subsequently, to reveal the temporal interaction among the firing of cell types and LFP features. Bottom, Fluoromicrograph of two recorded PCs (red; scale bar; 200 µm). ***B***, Top, Spike train of a PVBC (gray) and two subsequently recorded PCs (blue and green; same cells as in panel ***A***), each aligned to the last spike of the PVBC before the IED (red spike and asterisk). Note the abrupt onset of fast PC firing when the PVBC firing ceased. Bottom, Wavelet spectrogram averaged over *n* = 50 consecutive IED events from 5 slices (250 in total). Individual frequencies were aligned to the last spike of a PVBC (red line) and normalized by their averages. Note the emergence of fast-RIPs when PVBCs stop firing, characterized by a gradual decrease of their peak frequency throughout their course. ***C***, Top, Representative firing patterns of a firing type I PC during 50 consecutive IEDs, aligned to the PC spike closest to fast-RIP onset. Type I PCs were active only during the fast-RIP events, when PVBCs were silent, and their activity gradually declined during the IED. LFP of a single IED is shown along with the average fast-RIP power (black; individuals traces overlaid in gray, *n* = 50) below. Bottom, Same for a representative firing type II PC. Type II PCs started to become active before the fast-RIP, maintained their activity during, and stopped firing gradually after the fast-RIP oscillations. ***D***, Spike histograms of firing type I (top; *n* = 24) and firing type II PCs (middle; *n* = 8; histograms are clipped due to magnified ordinate). Note that firing type I PCs are only active during high fast-RIP power (indicated by gray shaded area), while firing type II PCs are active also outside of fast-RIPs (red arrows indicate these periods). Different colors in the type II plot represent individual cells, illustrating consistent bursting outside the fast-RIP period. ***E***, Firing type II PCs exhibited a gradual increase in activity before the onset of the IEDs, followed by a refractory period afterward (*n* = 8 cells; Pre-I vs II, *p* = 0.039; Pre-II vs Post-event, *p* = 0.0078; Wilcoxon signed-rank test). ***F***, A similar buildup of activity was observed in PVBCs, which exhibited a parallel increase in firing concurrent with that of bursting PCs (*n* = 7 cells; Pre-I vs II, *p* = 0.016; Pre-II vs Post-event, *p* = 0.016; Wilcoxon signed-rank test).

Ripple indexes were calculated by fitting a combination of a Gaussian and a sinusoidal function

$$y(t)\;=\;[a(\sin\;(\omega t)\;+\;b]\;∙\;e^{-{\left(t-\mu \right)}^{2}/2\tau^{2}},$$

to CCGs of PVBC cell pairs, where *t* is the autocorrelogram’s time variable and *a*, *b*, *ω*, *µ*, and *τ* are the fitted parameters. Here, *a* denotes the amplitude and *b* denotes the offset of the sinusoidal component, and their ratio was defined as the ripple index. For calculating spike histograms and average wavelet spectrograms in Figure 3*B*, the last spike of the loose-patch recorded PVBC before the IED peak was used as a triggering event. To calculate the firing rate of type II PCs and PVBCs in relation to IED onset, time windows were defined as Pre-I (−300 to −150 ms), II (−150 to −10 ms), and Post-event (+150 to +300 ms) from IED onset. In Figure 3*C*, fast-RIP onset, defined as the time point when ripple power crossed 5% of the maximal ripple power amplitude of the individual fast-RIP event, was used as a trigger. For the normalized PC spike histograms presented in Figure 3*D*, the fast-RIP start or the last PVBC spike was used as the triggering event, as indicated. Monte Carlo approximation of LFP oscillations based on PC cell bursting was performed by convolving a single, randomly selected IED-associated spike sequence from in vitro recorded PC bursts (*n* = ∼1,500 IED event associated bursts from 10 type I PCs) with a randomly drawn onset time from the PC spiking onset distribution (*n* = 700 events from 4 PC pairs), repeated 10,000 times. This simulation was repeated with the inclusion of type II PCs, reflecting the ratio of randomly sampled type I and type II cells in loose-patch recordings (Fig. 3*D*; *n* = ∼1,500 IED event associated bursts from 8 type II PCs). Intraburst interspike intervals during individual IED-associated bursts were randomly reordered to generate shuffled controls.

## Results

### Inhibition does not contribute to pathological fast-ripple generation

LFPs are largely generated by synaptic currents and action potentials, the relative contributions of which two components could differ substantially across network phenomena. We used local drug applications in acute hippocampal slices to dissect these generator mechanisms of both physiological ripples (RIP) and pathological fast-ripples (fast-RIP) in the CA3 area of the hippocampus.

Ionic mechanisms key to neural network functions can be manipulated by potent pharmacons, thereby separating distinct generators of the LFP. To avoid influencing large neuronal populations by global drug applications that may change network states and act epileptogenic, we used local drug injections only affecting a limited area of CA3. This was confirmed by placing an LFP electrode in the close vicinity of the glass capillary used for drug application, and a second one placed at ∼150 µm distance to exclude possible spillover effects (Fig. 1*A*; see also Materials and Methods).

First, we blocked local action potential (AP) generation by puffing a small amount of tetrodotoxin (TTX) focally into the CA3 pyramidal layer [0.1 µM, sufficient to block AP generation without affecting transmitter release, see Heifets et al. (2008) *n* = 7 slices; Fig. 1*B*]. This reversibly decreased ripple power (spectral power in the ripple frequency band, see Materials and Methods) together with a drop in MUA both during SWRs and IEDs (Fig. 1*B*–*D*, green, *n* = 7 slices). This decrease was more robust in the case of IEDs, suggesting that action potentials contribute prominently to IEDs and fast-RIP generation.

Next, in a complementary experiment, we tested the contribution of synaptic currents to the generation of RIP and fast-RIP oscillations. To address this, it was sufficient to block perisomatic inhibitory currents by local application of the GABA_A_R antagonist gabazine (10 µM, *n* = 7 slices), since there are no excitatory synapses in the perisomatic region (Megías et al., 2001). We found that ripple power during SWRs decreased robustly as shown previously (Schlingloff et al., 2014), suggesting that inhibitory synaptic currents contributed to RIP generation. However, gabazine did not cause detectable changes in fast-RIP power and MUA during IEDs (Fig. 1*B*–*D*). Thus, action potentials but not inhibitory currents contributed to the generation of fast-RIP currents during IEDs.

### Fast-ripples replace physiological ripples upon local elimination of inhibition

Blocking perisomatic inhibition focally first abolished physiological SWRs on the local electrode, but not on the distant electrode, as shown above (Fig. 1*B*,*E*, early phase of inj.). Then, in some cases (*n* = 3 slices), IEDs gradually appeared on the local electrode (Fig. 1*E*, late phase of inj., top), synchronously with the SWRs on the distant electrode (Fig. 1*E*, late phase of inj., bottom).

These IEDs were characterized by an absence of RIP power (Fig. 1*F*), while typical fast-RIP oscillations appeared in their late phase (Fig. 1*E*,*F*, late phase of inj., white arrowhead). In parallel, the distant electrode showed concurrent SWRs that were long in duration and featured two distinct peaks with normal RIP power. It is conceivable that the IED induced by the GABA_A_R blockade lasted longer than a typical SWR and conveyed excessive excitation that initiated a second SWR in the slice, leading to the double-peak appearance on the distant electrode. Of note, fast-RIP could not replace RIP at the distant electrode since the excitatory drive was balanced by intact perisomatic inhibition at the control site. Perisomatic inhibition recovered few minutes after the Gabazine puff, showed by the re-occurrence of physiological RIPs on both LFP electrodes (Fig. 1*F*, recovery).

### PVBCs are desynchronized during epileptiform activity

It was shown that RIP oscillations were generated by the interconnected network of PV-expressing basket cells, where their synchronization depended on mutual inhibitory synaptic connections and excitatory inputs (Schlingloff et al., 2014). However, it is unknown whether the physiological high-frequency synchronization of PVBCs is altered in epileptic conditions. Therefore, we examined whether the spike synchrony of PVBCs changed with the emergence of epileptic activity. We recorded pairs of spiking PVBCs (*n* = 8) in parallel with LFP measurement during SWR to IED transition induced by the elevated potassium model of epileptic activity (see Materials and Methods; Fig. 2*A*). PVBCs typically fired 3–5 action potentials during SWRs. In contrast, they displayed elevated firing rate during epileptic activity outside of IEDs and showed signs of depolarization block during the fast-RIP oscillations, confirming previous findings (Karlocai et al., 2014).

During physiological conditions (SWR activity under normal [K^+^]_e_), PVBC firing showed strong ripple modulation, revealed by the autocorrelation of single-cell spikes (Fig. 2*B*, left; *n* = 16 cells). In addition, spike cross-correlation of PVBC pairs recorded from two distinct locations (100–300 µm apart) also displayed prominent modulation at ripple frequency (Fig. 2*B*, right; *n* = 8 cell pairs), demonstrating ripple-related synchrony in the PVBC network. In contrast, the same PVBCs lost both regularity and synchrony of their firing during epileptic conditions (IED activity under elevated [K^+^]_e_), as shown by their altered auto- and cross-correlograms (Fig. 2*C*; *n* = 10 and 5, respectively). Ripple modulation index (Fig. 2*D*,*E*; see Materials and Methods) revealed complete loss of high-frequency synchronization between PVBC cell pairs under epileptic conditions.

### Spatially coherent ripples are replaced by desynchronized fast-RIPs during epileptiform activity

If the inhibitory network synchronization demonstrated above plays an essential role in RIP generation, then RIP oscillations are expected to be spatially more coherent than fast-RIPs due to the intact medium-range inhibitory synchronization that breaks down during epileptic activity. To test this prediction, we recorded LFP activity from the CA3 with two electrodes separated by about 300 µm and calculated wavelet coherence between the two LFPs during RIPs and fast-RIPs (Fig. 2*F*,*G*; *n* = 5 slices, 50 consecutive events in each slice). In each slice, we first recorded SWRs, which were subsequently turned into IEDs by pharmacological means (elevated [K^+^]_e_). Cross-wavelet spectra revealing shared frequency components among the two signals displayed strong shared RIP (∼200 Hz) and fast-RIP (≥200 Hz) components during SWRs and IEDs, respectively (Fig. 2*F*). Wavelet coherence, which reveals phase coupling independent of spectral power, indicated strong spatial coupling of sharp wave-associated RIPs (Fig. 2*G*, left), whereas there was no spatial coherence among fast-RIP oscillations (Fig. 2*G*, right).

### PCs fire bursts when inhibition collapses

Previous work of Karlocai et al. suggested a relationship among PVBC depolarization block, PC firing increase, and fast-RIP occurrence, based on correlated LFP and single-cell recordings (Karlocai et al., 2014). However, the relative timing between interneuronal firing and fast-RIP is unknown (Jiruska et al., 2017). Therefore, we measured the precise temporal relationship of PVBC and PC spiking during IEDs and fast-RIP occurrence by performing parallel multiple loose-patch and LFP recordings from the CA3 in acute hippocampal slices (Fig. 3*A*).

We calculated spike-triggered LFP average (STA) and spectrogram triggered on the last AP of the recorded PVBC before entering depolarization block (Fig. 3*B*, red asterisk). STA revealed that fast-RIP oscillation started abruptly at the time when PVBCs stopped firing (Fig. 3*B*, bottom).

Next, we aligned PC spiking to the same time point of the last PVBC spike, which revealed two distinct PC firing types. Most PCs (24/32, 75%) started to fire bursts exclusively when PVBCs stopped firing and fast-RIPs appeared (Fig. 3*B*–*D*, green, firing type I). A smaller subset of PCs (8/32, 25%) fired bursts before fast-RIP onset as well (Fig. 3*B*–*E*, blue, firing type II), although these bursts were shorter than those during fast-RIPs. The burst probability of these cells started to increase gradually before and showed refractoriness after the IEDs (Fig. 3*C*,*E*). Bursts that were not simultaneous with fast-RIPs were not associated with visible population events in the LFP, nor did they display synchrony among each other. Notably, PVBCs also displayed a gradual buildup of activity resembling that of type II PCs, suggesting reciprocal or coordinated dynamics between these interneurons and a subset of excitatory cells during the pre-fast-RIP phase (Fig. 3*F*). This parallel activation pattern may reflect an early preparatory phase of the network leading up to fast-RIP generation.

### Distinct epileptic and nonepileptic firing types correspond to superficial and deep CA3 pyramidal cells

In addition to the “classical” superficial CA3 pyramidal cells, Hunt et al. recently described another CA3 PC type, which largely lacks thorny excrescences along its proximal apical dendrites and display higher CSB probability (“athorny” or deep PCs; Hunt et al., 2018). This raised the possibility that the two fast-RIP-associated firing types identified above might correspond to these anatomically and physiologically distinct PC classes.

**Figure 4.**
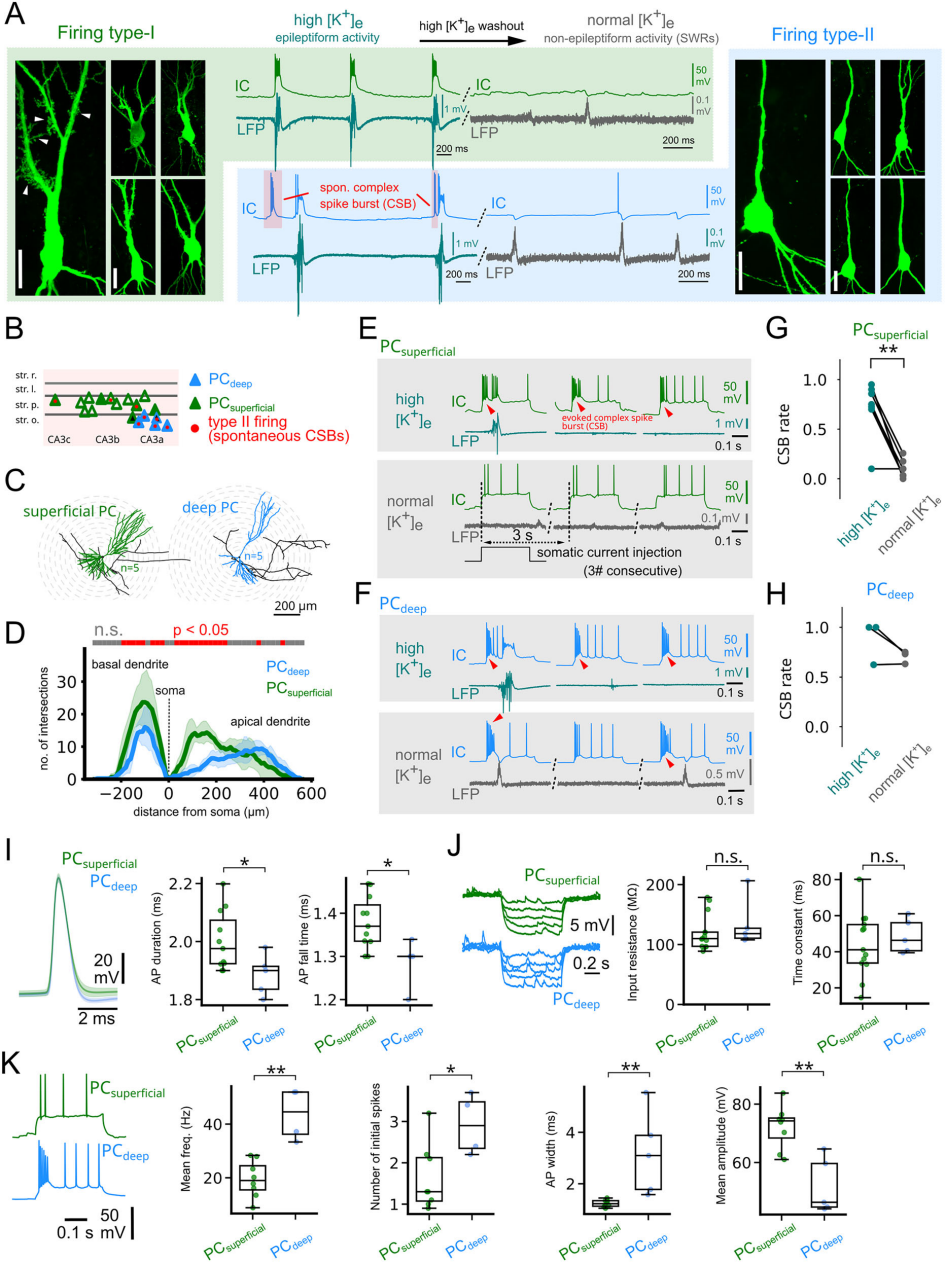
Distinct firing patterns correspond to deep and superficial CA3 pyramidal cells with unique physiological and anatomical properties. ***A***, Left, High magnification confocal image and corresponding intracellular recordings (middle, top; recorded at resting membrane potential) from a superficial PC. Smaller images show further superficial PC examples (scale bars, 20 µm). Right, Confocal image and corresponding intracellular recordings (middle, bottom) from a deep PC. Smaller images show other identified deep pyramidal cells. Epileptic and nonepileptic (SWR) activity is shown for both neurons (top, intracellular recording; bottom, LFP). Note that the superficial PC fired bursts during IEDs (firing type I), whereas the deep pyramidal cell fired spontaneous complex spike bursts (CSB) before epileptic events and single APs occasionally before SWRs as well (firing type II). ***B***, Layer distribution of the recorded CA3 PCs with their morphology and firing type indicated. PCs lacking proximal thorny excrescences were found in deeper layers of CA3a, consistently with Hunt et al. (2018). All anatomically identified deep PCs (*n* = 5 of 5) belonged to firing type II, whereas most superficial PCs showed firing type I (*n* = 10 of 13, filled triangles indicate reconstructed neurons, black-filled triangles specifically highlight example cells shown in panel ***C***). ***C***, Example of a reconstructed superficial (green) and deep PC (blue). Note the denser basal and proximal apical dendritic branching of the superficial PC. ***D***, Distribution of dendritic branching showed denser basal and proximal apical branching of superficial PCs (*n* = 5 for both types), confirming Hunt et al. (2018). Red segments on the horizontal line indicate significant differences in dendritic intersections between the two cell types (*p* = 0.014 in basal dendritic segment from −210 to −20 µm, *p* = 0.0014 in the apical dendritic segment from 40 to 250 µm, Mann–Whitney *U* test). ***E***, Top, Intracellular recordings of a superficial PC and corresponding LFP traces during somatic current injections (300 pA) under epileptiform conditions. Somatic current injections reliably evoked complex spike bursts. Bottom, Recordings of the same neuron during somatic current injections after washout of high-potassium ACSF (nonepileptiform condition) indicated a strong reduction of CSB firing. ***F***, The same as in ***E*** for an example deep PC. This cell fired CSBs both under epileptiform and nonepileptiform conditions. ***G***, Quantification of CSB occurrence in response to somatic current injections in superficial PCs under epileptiform and nonepileptiform conditions (SWRs). Most superficial PCs tested (*n* = 6 of 7) fired CSBs dominantly under the epileptiform conditions (median CSB rates with 1st and 3rd quartile during epileptiform state, 0.75 [0.71, 0.88]; nonepileptiform state, 0.1 [0.03, 0.13]; *p* = 0.006, Wilcoxon signed-rank test). ***H***, The same as in ***G*** for deep PCs. Deep pyramidal cells fired CSBs at similar rates under epileptiform and nonepileptiform conditions (*n* = 3 of 3; epileptiform, 1.0 [0.81, 1.0]; nonepileptiform, 0.73 [0.68, 0.74]; *p* = 0.51, Wilcoxon signed-rank test). ***I***, Action potential (AP) properties measured from isolated spontaneous APs. Representative traces (left) and quantification of AP duration and AP fall time revealed slightly broader spikes in superficial PCs. APs were analyzed outside of bursts to avoid confounds introduced by the progressive amplitude reduction and temporal broadening seen within bursts. ***J***, Passive membrane properties did not differ significantly between the two cell types. Representative voltage responses to hyperpolarizing current steps and quantification of input resistance and membrane time constant did not show statistical difference. ***K***, Firing properties in response to repeated depolarizing current steps: representative voltage traces (left) for deep (blue) and superficial (green) PCs, and quantification of mean firing frequency, number of initial spikes (i.e., the number of spikes within the first 10% of the current injection step), AP width, and its mean amplitude, all of which differed significantly between the two groups due to the presence of burst firing in deep PCs (*n* = 11 and 5 superficial and deep PCs, respectively).

To test this, we carried out firing type classification of CA3 PCs in loose-patch configuration and then performed intracellular recording and subsequent anatomical identification. We found that most of the anatomically identified superficial PCs (*n* = 10/13) showed type I firing, with their bursts restricted to the duration of fast-RIP oscillations (Fig. 4*A*, left). In contrast, all recorded deep PCs were characterized by type II firing, displaying bursts outside of fast-RIP periods (*n* = 5/5; Fig. 4*A*, right). These cells were found in the deep layers of the CA3a region and displayed long distal apical dendritic branches (Fig. 4*B*–*D*) as described before (Hunt et al., 2018). In line with more recent work (Balind et al., 2019), they possessed a few complex spines on the distal segment of their apical dendrite.

While superficial PCs show a low propensity for CSB in physiological conditions (Hunt et al., 2018), we wondered whether they could generate CSBs during epileptic state. We found that nearly all superficial PCs (*n* = 7 out of 8 neurons tested) responded with CSBs to somatic current injections during epileptic state (high [K^+^]_e_), similarly to deep PCs (*n* = 3 out of 3 neurons tested; Fig. 4*E*–*H*). Importantly, superficial PCs stopped firing CSBs when the slice returned to nonepileptic state after the washout of high [K^+^]_e_, unlike deep PCs that retained their ability to produce CSBs. To further assess whether type I and type II cells represent distinct neuronal subtypes, we compared their intrinsic electrophysiological properties. We found that superficial (type I) PCs exhibited significantly longer AP durations, slower repolarization (fall time), lower firing rates, and shorter initial spikes compared with deep (type II) PCs (Fig. 4*I*–*K*), supporting the notion that these groups differ not only in morphology and firing patterns but also in fundamental biophysical characteristics.

### Pseudosynchrony of PC firing arises from the simultaneous onset of stereotypic bursts

**Figure 5.**
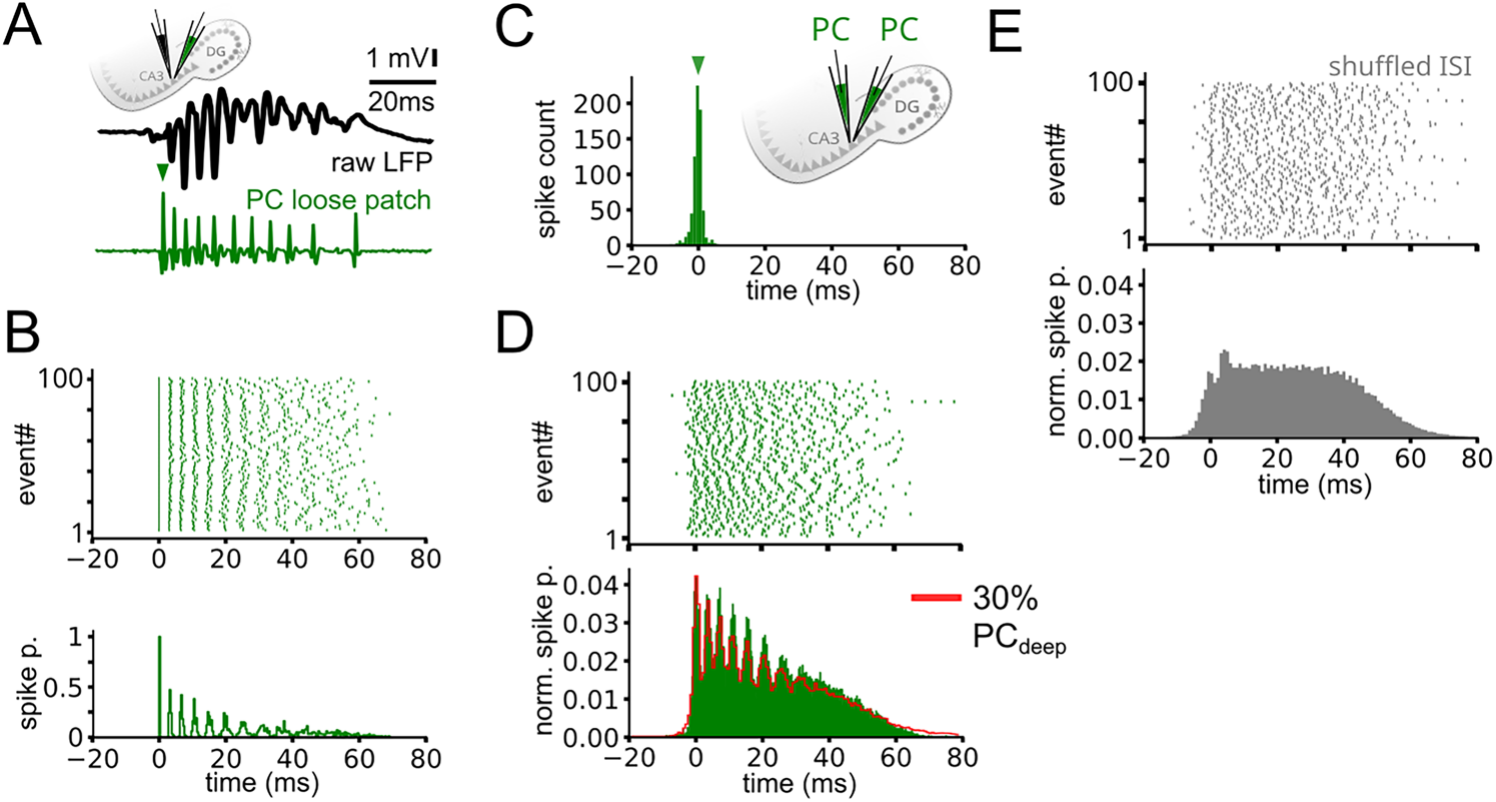
Fast-RIPs emerge from the pseudosynchronous firing of PCs. ***A***, Representative LFP trace of an IED and the firing of a local type I PC. ***B***, Raster plot and perievent time histogram (PETH; spike p., spike probability) of the same PC during 100 consecutive IEDs, aligned to the first spike during the IED. Note the highly stereotypic firing pattern, especially after the onset of the bursts. ***C***, Histogram of the time difference of first spikes of concurrently recorded type I PCs (inset, *n* = 4 pairs) during IEDs. Note that type I PCs started to fire near synchronously, within a narrow time window (mean ± SD, 0.24 ± 1.65 ms). ***D***, Population PETH of simulated pyramidal cells that started their stereotypic bursts nearly simultaneously (spike time differences of first spikes drawn from distribution in ***C***, Monte Carlo simulation, see Materials and Methods), demonstrating the emergence of high-frequency periodic activity. Note that inclusion of deep PCs, which showed less stereotypic bursting patterns during IEDs, tended to decrease the amplitude of the high-frequency periodic activity. The proportion of deep PCs was set to 30% to match realistic cell type distributions based on our recordings. ***E***, Raster plot and PETH of simulated PCs with the intraburst interspike intervals shuffled.

Next, we explored how the rhythmicity of fast-RIP oscillations can arise from the assumed cacophony of pyramidal cell bursts when inhibition breaks down. We observed that most PCs showed type I firing and produced highly stereotypical bursts during subsequent IEDs (Fig. 5*A*,*B*). These stereotypical bursts started within a narrow time window (corresponding to the collapse of inhibition; Fig. 2*B*) for concurrently recorded nearby (<50 µm distance) PCs (Fig. 5*C*).

To test whether stereotypic bursts that start simultaneously can result in the observed high-frequency field signature of fast-RIPs, we convolved the stereotypical burst patterns (Fig. 5*B*) of multiple type I PCs with their onset coupling (Fig. 5*C*). We found that the resulting population firing distribution (Fig. 5*D*) strongly resembled the LFP recordings during fast-RIPs (Fig. 5*A*), which remained true after adding type II PC bursts to the simulation. This pattern was eliminated by shuffling the intraburst interspike intervals (Fig. 5*E*), suggesting that fast-RIP oscillations arise due to the pseudosynchrony of simultaneously initiated stereotypical PC bursts.

### Burst properties of pyramidal cells control fast-RIP oscillation characteristics

**Figure 6.**
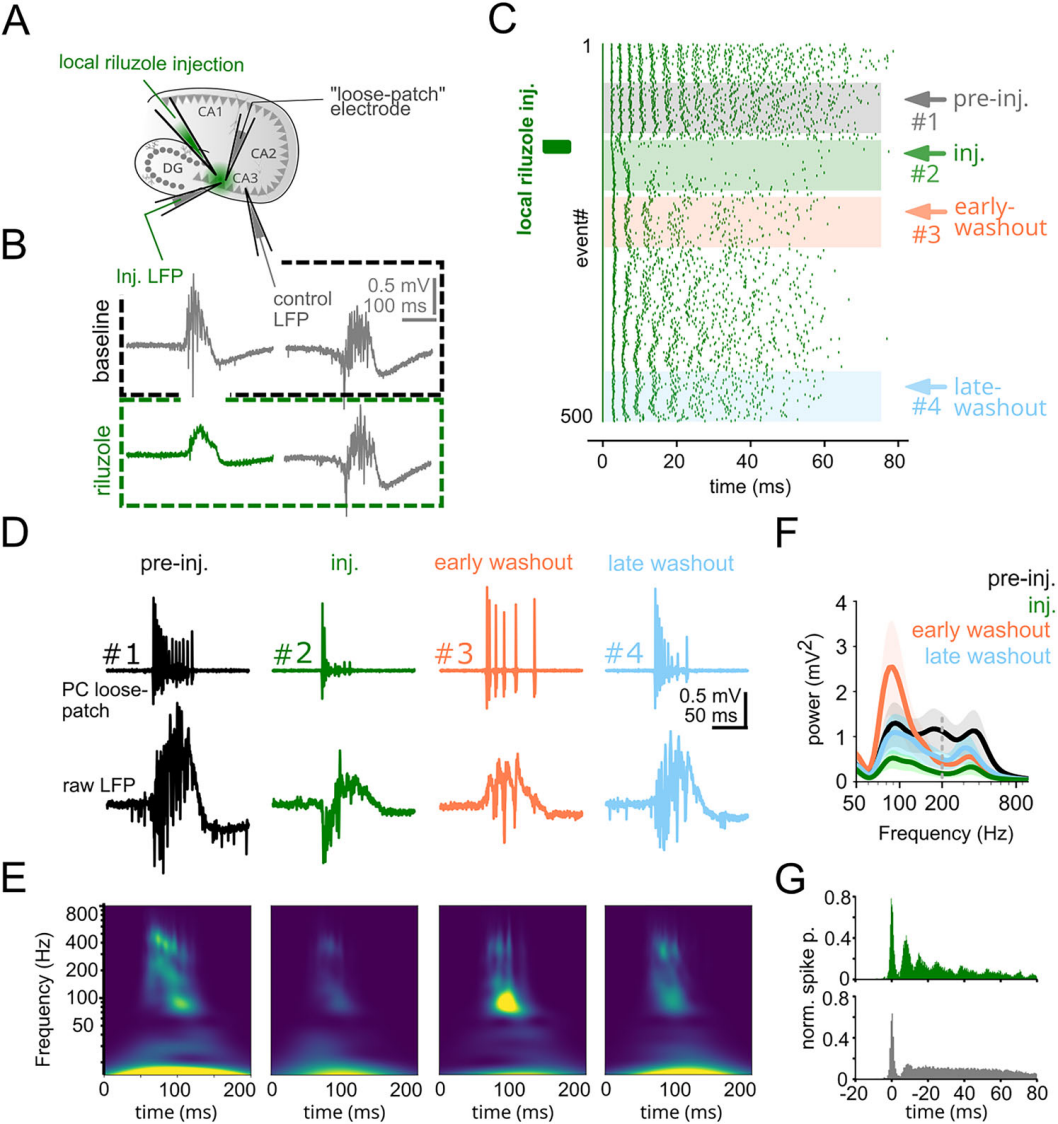
Modulation of fast-RIP oscillations via local modulation of pyramidal bursting. ***A***, Schematic of the experimental configuration. To prove that fast-RIP oscillations were causally related to the pseudosynchronous stereotypic PC bursts, we recorded LFP at two positions (∼150 µm) as well as PCs in cell-attached configuration during IEDs. A puff of the state-dependent sodium channel blocker riluzole (50 µM) was applied focally in the vicinity of one LFP electrode (“local electrode”) and the loose-patch recording electrode to modulate PC burst properties. ***B***, The puff eliminated fast-RIP oscillations from the local electrode (green), leaving the slow component of the IED intact. The LFP on the other (“distant”) electrode remained unchanged (gray). ***C***, Spike rasters of an example PC at different times before (#1), during (#2), and after (#3 and #4) the riluzole puff. Note that the stereotypic bursts were reduced to a few spikes upon the application (green box) of the puff (#2). During washout, a slower bursting with gradually increasing number of spikes returned (#3–4). ***D***, Top, Examples of PC spike trains at four different time points. Bottom, Corresponding IEDs recorded on the local electrode. The complexity and fast-RIP content of the LFP closely reflected the firing structure of the concurrently recorded PC. ***E***, Average wavelet spectrograms of the IEDs during the same four phases of Riluzole injection (each averaged over 50 consecutive IEDs). Riluzole robustly decreases all spectral components above about 50 Hz (second plot), followed by the recovery of slower (around 80 Hz, third plot), and then faster frequencies (fourth plot) during washout. ***F***, Average spectral power during the four phases of riluzole injection. Spectral components were divided to low and high frequencies for comparison. Average low-frequency (50–200 Hz) spectral power in phases 1, 2, 3, and 4 in mV^2^: 0.89 ± 0.33, 0.28 ± 0.13, 1.17 ± 0.72, 0.66 ± 0.34, respectively (1 vs 2, *p* < 0.001; 2 vs 3, *p* < 0.001; 3 vs 4, *p* < 0.001, Wilcoxon rank-sum test). Average high-frequency (200–500 Hz) spectral power in phases 1, 2, 3, and 4 in mV^2^: 0.98 ± 0.25, 0.21 ± 0.05, 0.40 ± 0.15, 0.54 ± 0.30, respectively (1 vs 2, *p* < 0.001; 2 vs 3, *p* < 0.01; 3 vs 4, *p* = 0.04, Wilcoxon rank-sum test). ***G***, Top, Monte Carlo simulation using spike time differences of first spikes drawn from the distribution in Figure 5*C* and altered burst firing patterns drawn from recordings in phase #3 showed an altered population PETH compared with panel Figure 5*D*. Bottom, shuffling of intraburst interspike intervals resulted in an aperiodic PETH due to the abolished firing stereotypy.

To causally test whether stereotypic PC firing controls the rhythmicity of fast-RIP oscillations, we performed local application of riluzole (50 µM; Fig. 6*A*), an antiepileptic drug (Diao et al., 2013) that alters sodium channel availability in a state-dependent manner (Benoit and Escande, 1991), thus changing PC burst firing. We recorded LFP and the firing pattern of individual PCs at the site of drug application, as well as LFP at a more distant (control) site.

Short puff application of riluzole (*n* = 4, see Methods) immediately attenuated IEDs and suppressed fast-RIP oscillations on the local but not on the distant electrode (Fig. 6*B*), confirming that the drug effect was focal. Prior to riluzole application, PCs displayed stereotypical burst patterns during fast-RIP oscillations recorded on the local LFP electrode (*n* = 3; Fig. 6*C*,*D*, #1). Upon short puff application of riluzole, PC burst firing was strongly attenuated, along with a blockade of fast-RIPs (Fig. 6*C*,*D*, #2). During washout of the drug, PCs started to fire bursts of increasing length with a low intraburst frequency. These low-frequency bursts still showed remarkable stereotypy across consecutive events (Fig. 6*C*,*D*, #3). In parallel, fast-RIP oscillations displayed a concordant low frequency (Fig. 6*E*,*F*). Performing the same convolution-based analysis of PC firing as in Figure 5*D*,*E* reproduced the spectral characteristics of these low-frequency fast-RIPs (Fig. 6*G*). Characteristic PC firing patterns recovered with gradual washout of riluzole (Fig. 6*C*,*D*, #4), with a concomitant recovery of predrug fast-RIP oscillations.

These results suggest that fast-RIP oscillations, in contrast with sharp wave-associated ripples, do not arise from oscillatory network interactions but from stereotypical PC bursting initiated by the collapse of perisomatic inhibition that results in pseudosynchrony. Therefore, physiological and pathological ripples are distinct forms of high-frequency oscillations, generated by different mechanisms. Nonetheless, the identification of two distinct fast-RIP-associated firing patterns by superficial and deep CA3 PCs suggests that deep PCs might be important in the initiation of IEDs, similar to that of SWRs (Hunt et al., 2018).

## Discussion

Computational studies suggested that as oscillatory frequency increases, the contribution of synaptic potentials to LFP generation weakens (Schomburg et al., 2012; Fink et al., 2015). In agreement with the above, by using local pharmacological manipulations in vitro, we demonstrated that action potentials but not inhibitory currents contribute to fast-RIP generation (Fig. 1), opposed to RIPs where LFP generation is dominated by synchronous IPSPs (Schlingloff et al., 2014; Stark et al., 2014; Gan et al., 2017). Furthermore, local elimination of inhibition during SWRs transformed physiological ripples to fast-RIPs (Fig. 1). These results suggest that hyperexcitability and the breakdown of inhibition are localized rather than uniform in cases of physiological and pathological ripple co-occurrence in vivo—i.e., only certain “patches” of the network become hypersynchronized while neighboring regions retain enough inhibition to generate normal SWRs. A similar phenomenon has also been observed in vivo following local GABA_A_R blocker injection (Stark et al., 2014). Patchy degeneration of inhibition (or focal interneuron loss) can create small hyperexcitable clusters that produce fast-ripples, while adjacent areas with intact circuitry continue to support physiological ripples. Processes in the high-K^+^ model likely reflect what happens within those local epileptic microdomains. Our results further suggest that periods of activity associated with high excitatory gain (Buzsáki, 2006) such as SWRs or even strong synchronized extrahippocampal (e.g., entorhinal or dentate gyrus) inputs or microseizures can transiently produce conditions favoring fast-ripple generation in these microdomains, potentially impairing memory consolidation (Valero et al., 2017; Ewell et al., 2019).

**Figure 7.**
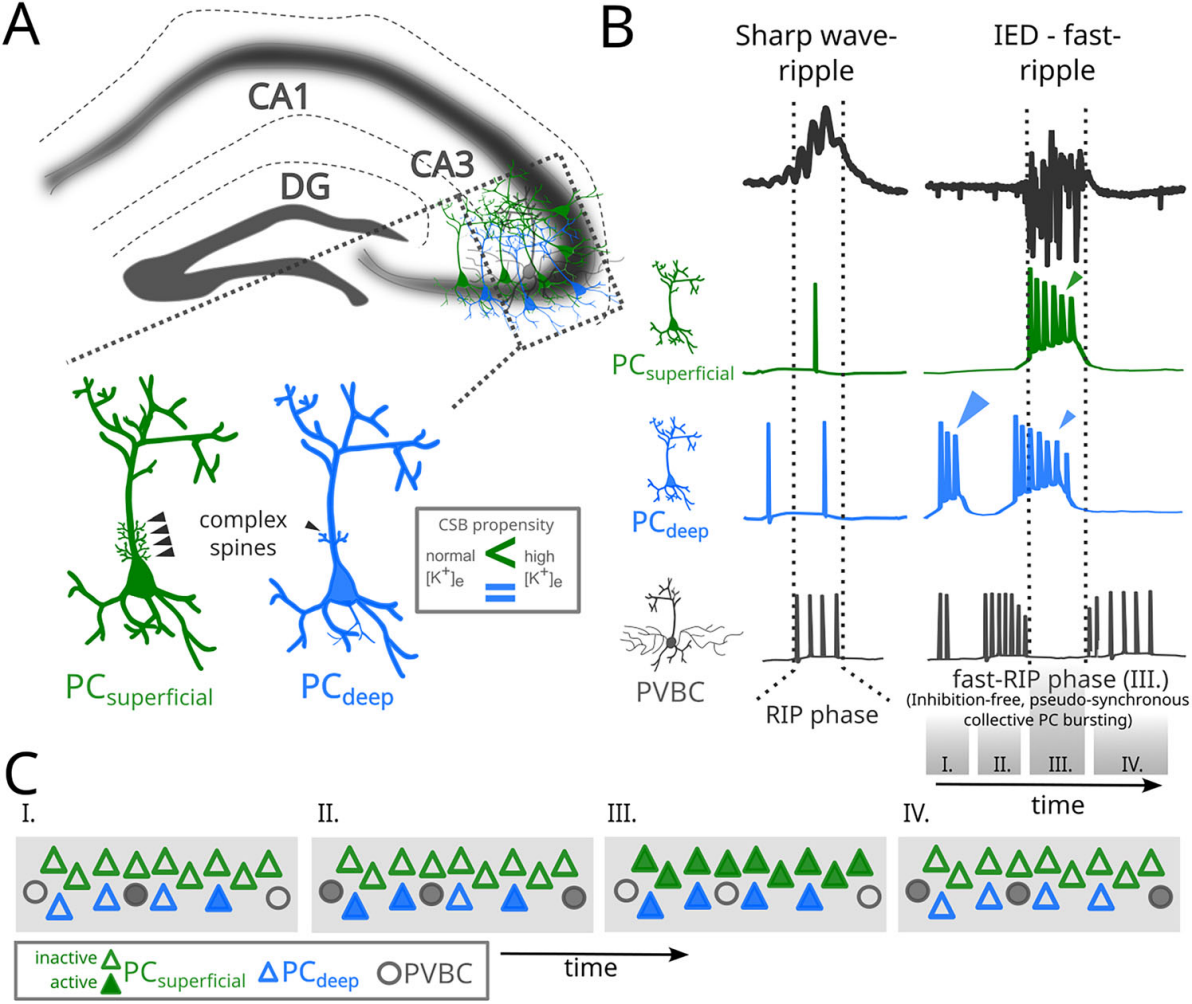
Summary mechanisms underlying physiological ripples and pathological fast-ripples in the CA3 hippocampal circuit. ***A***, Schematic of the hippocampal CA3 region, highlighting two distinct subtypes of pyramidal cells: superficial PCs (PC_superficial,_ green) and deep PCs (PC_deep_, blue). Both subtypes contribute to local network activity through distinct burst dynamics. Insets show morphological differences, including the presence of complex spines on superficial PC dendrites. ***B***, Electrophysiological signatures of sharp wave-ripples (left) and IED-fast-ripple events (right). SWRs are dominated by synchronous inhibitory postsynaptic potentials (IPSPs), involving coordinated activity of PV-expressing basket cells (PVBCs), while fast-ripples arise during PVBC depolarization block and are driven by pseudosynchronous action potentials of bursting PCs. Voltage traces from representative deep and superficial PCs and PVBCs show the transition from ripple to fast-ripple associated firing. ***C***, Schematic illustration of PC burst dynamics during epileptiform activity. I: Baseline state. II: Progressive increase in spontaneous bursting of deep pyramidal cells (type II), accompanied by a rise in PVBC activity. III: PVBC depolarization block enables the emergence of inhibition-free fast-ripples driven by stereotypic bursting. Concurrent pseudosynchronous bursting of superficial PCs (type I) and deep PCs give rise to fast-RIPs. IV: Collective bursting of PCs ceases. Simultaneously, PVBCs recover from depolarization block.

We also examined the temporal relationships among PC bursting (de la Prida et al., 2006; Foffani et al., 2007; Ibarz et al., 2010; Bragin et al., 2011), PVBC depolarization block (Karlocai et al., 2014), and fast-RIP occurrence during epileptiform activity. We found that fast-RIPs emerge at the time point when PVBCs stop firing and most pyramidal cells (firing type I) start to fire in bursts, coinciding with the fast-RIP phase (Fig. 3). We also found another, smaller pyramidal cell population (firing type II), the burst probability of which started to gradually increase before IEDs, making them a candidate for the source of the stochastic buildup of excitatory synaptic activity observed before IED onset (de la Prida et al., 2006). These spontaneously occurring CSBs in type II firing PCs could drive the CA3 recurrent network effectively, leading to higher excitatory gain and over-excitation of PVBCs (Fig. 3). This notion was also supported by ramping PVBC activity that paralleled that of type II PC CSBs. In turn, the depolarization block of PVBCs marks the start of an inhibition-free fast-RIP phase characterized by collective PC bursting (Figs. 3–5). A schematic summary of these events is provided in Figure 7.

Post hoc anatomical analysis revealed that cells with bursts restricted to the fast-RIP event (type I) were “classical” superficial CA3 PCs. In contrast, spontaneously bursting cells (type II) mostly belonged to the recently discovered deep CA3 PC type (Hunt et al., 2018). Although CSB is physiologically a feature characteristic to deep PCs, we showed that superficial PCs develop CSB capability under epileptic conditions (Fig. 4). A key factor in this process is the extracellular potassium concentration (K^+^_e_), which is known to rise during seizures (Fröhlich et al., 2008). Increasing evidence suggests that elevated K^+^_e_ not only accompanies seizures but also actively drives their initiation and propagation. This occurs through multiple mechanisms, including potassium transporter activity—such as the Na^+^/K^+^ ATPase (Sun et al., 2022), KCC2, and NKCC1 (Liu et al., 2020)—as well as passive uptake through astrocytic inward-rectifying K^+^ (Kir) channels (Villa and Combi, 2016; Ohno et al., 2021). Impaired potassium clearance has been linked to focal epileptic activity (David et al., 2009), while dysfunction of the KCC2 cotransporter promoted epileptic oscillations (Buchin et al., 2016). Notably, enhanced potassium buffering was shown to attenuate seizures (Zhao et al., 2022). Our findings suggest that elevated K^+^_e_ levels facilitate epileptic activity by enhancing the ability of both deep and superficial pyramidal cells to generate complex spike bursts. Crucially, this process depends on dendritic calcium spikes, which are tightly regulated by potassium channels (Balind et al., 2019; Kis et al., 2024). These results highlight a potential mechanistic link between extracellular potassium dynamics and seizure-related network excitability.

Local pharmacological manipulation of PC burst patterns (Fig. 6) revealed a causal relationship between the simultaneous onset of PC bursting and the concurrent fast-RIP oscillations. The oscillatory LFP pattern was generated by a mechanism termed pseudosynchrony, in which cofiring is simply caused by the precisely time locked onset of stereotypic PC bursts (Fig. 3). Thus, the main contributors to fast-RIP LFP oscillations were pseudosynchronous action potentials of bursting PCs, with no involvement of oscillatory inhibitory potentials, in sharp contrast with SWRs (Figs. 1, 2.). Our data support theoretical work demonstrating that fast-RIPs can be generated without involving network synchronization mechanisms when ensembles of biophysically similar cells are driven by a strong shared excitatory input (Gliske et al., 2017). We propose that LFP spectral components higher than individual PC firing rates could arise from the out-of-phase firing of spontaneously bursting PCs or a subset of cells possessing weaker or simply different spike stereotypy during fast-RIPs.

An independent consequence of the abrupt loss of perisomatic inhibition is the loss of mid-range spatial synchrony. As shown previously, PVBCs orchestrate physiological ripple frequency synchrony in vitro (Schlingloff et al., 2014) and in vivo (Stark et al., 2014). We showed that SWR-associated synchrony within the interconnected PVBC network breaks down during epileptiform activity. Along with this, the mid-range spatial synchrony of ripples degrades during fast-RIPs (Fig. 2), in agreement with a previous finding that as fast-RIP activity accelerates mid-range LFP cross-correlation strength decreases (Foffani et al., 2007).

Our findings showing that the spectral components and the spatial coherence of fast-RIPs, arising at loci with compromised inhibition, are significantly different from those of normal RIPs, suggest that these features have diagnostic value. Thus, analysis of high-frequency EEG components and their coherence might help delineate locations that are the primary sources of uncontrolled excitatory activity.

## References

[B1] Akam T, Kullmann DM (2014) Oscillatory multiplexing of population codes for selective communication in the mammalian brain. Nat Rev Neurosci 15:111–122. 10.1038/nrn3668 24434912 PMC4724886

[B2] Balind SR, Magó Á, Ahmadi M, Kis N, Varga-Németh Z, Lőrincz A, Makara JK (2019) Diverse synaptic and dendritic mechanisms of complex spike burst generation in hippocampal CA3 pyramidal cells. Nat Commun 10:1859. 10.1038/s41467-019-09767-w 31015414 PMC6478939

[B3] Behrens C, van den Boom L, de Hoz L, Friedman A, Heinemann U (2005) Induction of sharp wave-ripple complexes in vitro and reorganization of hippocampal networks. Nat Neurosci 8:1560–1567. 10.1038/nn1571 16222227

[B4] Benoit E, Escande D (1991) Riluzole specifically blocks inactivated Na channels in myelinated nerve fibre. Pflugers Arch 419:603–609. 10.1007/BF00370302 1664937

[B5] Birk N, Schönberger J, Somerlik-Fuchs KH, Schulze-Bonhage A, Jacobs J (2021) Ictal occurrence of high-frequency oscillations correlates with seizure severity in a rat model of temporal lobe epilepsy. Front Hum Neurosci 15:624620. 10.3389/fnhum.2021.624620 34168542 PMC8217452

[B6] Bragin A, Benassi SK, Kheiri F, Engel J (2011) Further evidence that pathological high frequency oscillations are bursts of population spikes derived from recordings of identified cells in dentate gyrus. Epilepsia 52:45–52. 10.1111/j.1528-1167.2010.02896.x PMC3057512 21204820

[B7] Buchin A, Chizhov A, Huberfeld G, Miles R, Gutkin BS (2016) Reduced efficacy of the KCC2 cotransporter promotes epileptic oscillations in a subiculum network model. J Neurosci 36:11619–11633. 10.1523/JNEUROSCI.4228-15.2016 27852771 PMC6231544

[B8] Buzsáki G (1989) Two-stage model of memory trace formation: a role for “noisy” brain states. Neuroscience 31:551–570. 10.1016/0306-4522(89)90423-5 2687720

[B9] Buzsáki G (2006) Rhythms of the brain. Oxford: Oxford Univ. Press.

[B10] Buzsáki G (2015) Hippocampal sharp wave-ripple: a cognitive biomarker for episodic memory and planning. Hippocampus 25:1073–1188. 10.1002/hipo.22488 26135716 PMC4648295

[B11] Buzsáki G, Chrobak JJ (1995) Temporal structure in spatially organized neuronal ensembles: a role for interneuronal networks. Curr Opin Neurobiol 5:504–510. 10.1016/0959-4388(95)80012-3 7488853

[B12] Buzsáki G, Leung LW, Vanderwolf CH (1983) Cellular bases of hippocampal EEG in the behaving rat. Brain Res 287:139–171. 10.1016/0165-0173(83)90037-1 6357356

[B13] David Y, Cacheaux LP, Ivens S, Lapilover E, Heinemann U, Kaufer D, Friedman A (2009) Astrocytic dysfunction in epileptogenesis: consequence of altered potassium and glutamate homeostasis? J Neurosci 29:10588–10599. 10.1523/JNEUROSCI.2323-09.2009 19710312 PMC2875068

[B14] de la Prida L, Huberfeld G, Cohen I, Miles R (2006) Threshold behavior in the initiation of hippocampal population bursts. Neuron 49:131–142. 10.1016/j.neuron.2005.10.034 16387645

[B15] Diao L, Hellier JL, Uskert-Newsom J, Williams PA, Staley KJ, Yee AS (2013) Diphenytoin, riluzole and lidocaine: three sodium channel blockers, with different mechanisms of action, decrease hippocampal epileptiform activity. Neuropharmacology 73:48–55. 10.1016/j.neuropharm.2013.04.057 23707481 PMC3766392

[B16] Draguhn A, Traub R, Schmitz D, Jefferys J (1998) Electrical coupling underlies high-frequency oscillations in the hippocampus in vitro. Nature 394:189–192. 10.1038/28184 9671303

[B17] Engel J, Bragin A, Staba R, Mody I (2009) High-frequency oscillations: what is normal and what is not? Epilepsia 50:598–604. 10.1111/j.1528-1167.2008.01917.x 19055491

[B18] Evangelista R, Cano G, Cooper C, Schmitz D, Maier N, Kempter R (2020) Generation of sharp wave-ripple events by disinhibition. J Neurosci 40:7811–7836. 10.1523/JNEUROSCI.2174-19.2020 32913107 PMC7548694

[B19] Ewell LA, Fischer KB, Leibold C, Leutgeb S, Leutgeb JK (2019) The impact of pathological high-frequency oscillations on hippocampal network activity in rats with chronic epilepsy. eLife 8:e42148. 10.7554/eLife.42148 30794155 PMC6386518

[B20] Fink CG, Gliske S, Catoni N, Stacey WC (2015) Network mechanisms generating abnormal and normal hippocampal high-frequency oscillations: a computational analysis. eNeuro 2:ENEURO.0024-15.2015. 10.1523/ENEURO.0024-15.2015 PMC4487885 26146658

[B21] Foffani G, Uzcategui Y, Gal B, Menendez de la Prida L (2007) Reduced spike-timing reliability correlates with the emergence of fast ripples in the rat epileptic hippocampus. Neuron 55:930–941. 10.1016/j.neuron.2007.07.040 17880896

[B22] Foster DJ, Wilson MA (2006) Reverse replay of behavioural sequences in hippocampal place cells during the awake state. Nature 440:680–683. 10.1038/nature04587 16474382

[B23] Fröhlich F, Bazhenov M, Iragui-Madoz V, Sejnowski TJ (2008) Potassium dynamics in the epileptic cortex: new insights on an old topic. Neuroscientist 14:422–433. 10.1177/1073858408317955 18997121 PMC2854295

[B24] Gan J, Weng S, Pernía-Andrade AJ, Csicsvari J, Jonas P (2017) Phase-locked inhibition, but not excitation, underlies hippocampal ripple oscillations in awake mice in vivo. Neuron 93:308–314. 10.1016/j.neuron.2016.12.018 28041883 PMC5263253

[B25] Gliske SV, Stacey WC, Lim E, Holman KA, Fink CG (2017) Emergence of narrowband high frequency oscillations from asynchronous, uncoupled neural firing. Int J Neural Syst 27:1650049. 10.1142/S0129065716500490 27712456 PMC5101151

[B26] Grinsted A, Moore JC, Jevrejeva S (2004) Application of the cross wavelet transform and wavelet coherence to geophysical time series. Nonlinear Process Geophys 11:561–566. 10.5194/npg-11-561-2004

[B27] Hájos N, Ellender TJ, Zemankovics R, Mann EO, Exley R, Cragg SJ, Freund TF, Paulsen O (2009) Maintaining network activity in submerged hippocampal slices: importance of oxygen supply. Eur J Neurosci 29:319–327. 10.1111/j.1460-9568.2008.06577.x 19200237 PMC2695157

[B28] Heifets BD, Chevaleyre V, Castillo PE (2008) Interneuron activity controls endocannabinoid-mediated presynaptic plasticity through calcineurin. Proc Natl Acad Sci U S A 105:10250–10255. 10.1073/pnas.0711880105 18632563 PMC2481322

[B29] Hunt DL, Linaro D, Si B, Romani S, Spruston N (2018) A novel pyramidal cell type promotes sharp-wave synchronization in the hippocampus. Nat Neurosci 21:985–995. 10.1038/s41593-018-0172-7 29915194

[B30] Ibarz JM, Foffani G, Cid E, Inostroza M, de la Prida LM (2010) Emergent dynamics of fast ripples in the epileptic hippocampus. J Neurosci 30:16249–16261. 10.1523/JNEUROSCI.3357-10.2010 21123571 PMC6634823

[B31] Jadhav SP, Rothschild G, Roumis DK, Frank LM (2016) Coordinated excitation and inhibition of prefrontal ensembles during awake hippocampal sharp-wave ripple events. Neuron 90:113–127. 10.1016/j.neuron.2016.02.010 26971950 PMC4824654

[B32] Jiruska P, Alvarado-Rojas C, Schevon CA, Staba R, Stacey W, Wendling F, Avoli M (2017) Update on the mechanisms and roles of high-frequency oscillations in seizures and epileptic disorders. Epilepsia 58:1330–1339. 10.1111/epi.13830 28681378 PMC5554080

[B33] Karlocai MR, Kohus Z, Kali S, Ulbert I, Szabo G, Mate Z, Freund TF, Gulyas AI (2014) Physiological sharp wave-ripples and interictal events in vitro: what’s the difference? Brain 137:463–485. 10.1093/brain/awt348 24390441

[B34] Khodagholy D, Gelinas JN, Buzsáki G (2017) Learning-enhanced coupling between ripple oscillations in association cortices and hippocampus. Science 358:369–372. 10.1126/science.aan6203 29051381 PMC5872145

[B35] Kis N, Lükő B, Herédi J, Magó Á, Erlinghagen B, Ahmadi M, Raus Balind S, Irás M, Ujfalussy BB, Makara JK (2024) Cholinergic regulation of dendritic Ca^2+^ spikes controls firing mode of hippocampal CA3 pyramidal neurons. Proc Natl Acad Sci U S A 121:e2321501121. 10.1073/pnas.2321501121 39503887 PMC11572977

[B36] Kramer MA, et al. (2019) Scalp recorded spike ripples predict seizure risk in childhood epilepsy better than spikes. Brain 142:1296–1309. 10.1093/brain/awz059 30907404 PMC6487332

[B37] Liu AA, et al. (2022) A consensus statement on detection of hippocampal sharp wave ripples and differentiation from other fast oscillations. Nat Commun 13:6000. 10.1038/s41467-022-33536-x 36224194 PMC9556539

[B38] Liu R, Wang J, Liang S, Zhang G, Yang X (2020) Role of NKCC1 and KCC2 in epilepsy: from expression to function. Front Neurol 10:1407. 10.3389/fneur.2019.01407 32010056 PMC6978738

[B39] Maier N, Morris G, Schuchmann S, Korotkova T, Ponomarenko A, Böhm C, Wozny C, Schmitz D (2012) Cannabinoids disrupt hippocampal sharp wave-ripples via inhibition of glutamate release. Hippocampus 22:1350–1362. 10.1002/hipo.20971 21853502

[B40] Maier N, Nimmrich V, Draguhn A (2003) Cellular and network mechanisms underlying spontaneous sharp wave-ripple complexes in mouse hippocampal slices. J Physiol 550:873–887. 10.1113/jphysiol.2003.044602 12807984 PMC2343079

[B41] McNaughton BL, Douglas RM, Goddard GV (1978) Synaptic enhancement in fascia dentata: cooperativity among coactive afferents. Brain Res 157:277–293. 10.1016/0006-8993(78)90030-6 719524

[B42] Megías M, Emri Z, Freund TF, Gulyás AI (2001) Total number and distribution of inhibitory and excitatory synapses on hippocampal CA1 pyramidal cells. Neuroscience 102:527–540. 10.1016/s0306-4522(00)00496-6 11226691

[B43] Meyer AH, Katona I, Blatow M, Rozov A, Monyer H (2002) In vivo labeling of parvalbumin-positive interneurons and analysis of electrical coupling in identified neurons. J Neurosci 22:7055–7064. 10.1523/JNEUROSCI.22-16-07055.2002 12177202 PMC6757887

[B44] Nádasdy Z, Hirase H, Czurkó A, Csicsvari J, Buzsáki G (1999) Replay and time compression of recurring spike sequences in the hippocampus. J Neurosci 19:9497–9507. 10.1523/JNEUROSCI.19-21-09497.1999 10531452 PMC6782894

[B45] Ohno Y, Kunisawa N, Shimizu S (2021) Emerging roles of astrocyte Kir4.1 channels in the pathogenesis and treatment of brain diseases. IJMS 22:10236. 10.3390/ijms221910236 34638578 PMC8508600

[B46] Oliva A, Fernández-Ruiz A, Buzsáki G, Berényi A (2016) Role of hippocampal CA2 region in triggering sharp-wave ripples. Neuron 91:1342–1355. 10.1016/j.neuron.2016.08.008 27593179 PMC8138857

[B47] Ranjan R, Van Geit W, Moor R, Rössert C, Riquelme JL, Damart T, Jaquier A, Tuncel A, Mandge D, Kilic I (2024) eFEL. Available at: https://zenodo.org/doi/10.5281/zenodo.593869 [Accessed July 8, 2025].

[B48] Santana-Gomez CE, Engel J Jr, Staba R (2022) Drug-resistant epilepsy and the hypothesis of intrinsic severity: what about the high-frequency oscillations? Epilepsia Open 7:S59–S67. 10.1002/epi4.12551 34861102 PMC9340307

[B49] Schlingloff D, Káli S, Freund TF, Hájos N, Gulyás AI (2014) Mechanisms of sharp wave initiation and ripple generation. J Neurosci 34:11385–11398. 10.1523/JNEUROSCI.0867-14.2014 25143618 PMC6615505

[B50] Schomburg EW, Anastassiou CA, Buzsaki G, Koch C (2012) The spiking component of oscillatory extracellular potentials in the rat hippocampus. J Neurosci 32:11798–11811. 10.1523/JNEUROSCI.0656-12.2012 22915121 PMC3459239

[B51] Stark E, Roux L, Eichler R, Senzai Y, Royer S, Buzsáki G (2014) Pyramidal cell-interneuron interactions underlie hippocampal ripple oscillations. Neuron 83:467–480. 10.1016/j.neuron.2014.06.023 25033186 PMC4393648

[B52] Sun J, Zheng Y, Chen Z, Wang Y (2022) The role of Na^+^ -K^+^ - ATPase in the epileptic brain. CNS Neurosci Ther 28:1294–1302. 10.1111/cns.13893 35751846 PMC9344081

[B53] Tamilia E, Matarrese MAG, Ntolkeras G, Grant PE, Madsen JR, Stufflebeam SM, Pearl PL, Papadelis C (2021) Noninvasive mapping of ripple onset predicts outcome in epilepsy surgery. Ann Neurol 89:911–925. 10.1002/ana.26066 33710676 PMC8229023

[B54] Torrence C, Compo GP (1998) A practical guide to wavelet analysis. Bull Am Meteorol Soc 79:61–78. 10.1175/1520-0477(1998)079<0061:APGTWA>2.0.CO;2

[B55] Trevelyan AJ, Sussillo D, Watson BO, Yuste R (2006) Modular propagation of epileptiform activity: evidence for an inhibitory veto in neocortex. J Neurosci 26:12447–12455. 10.1523/JNEUROSCI.2787-06.2006 17135406 PMC6674895

[B56] Valero M, Averkin RG, Fernandez-Lamo I, Aguilar J, Lopez-Pigozzi D, Brotons-Mas JR, Cid E, Tamas G, Menendez de la Prida L (2017) Mechanisms for selective single-cell reactivation during offline sharp-wave ripples and their distortion by fast ripples. Neuron 94:1234–1247.e7. 10.1016/j.neuron.2017.05.032 28641116

[B57] Vaz AP, Inati SK, Brunel N, Zaghloul KA (2019) Coupled ripple oscillations between the medial temporal lobe and neocortex retrieve human memory. Science 363:975–978. 10.1126/science.aau8956 30819961 PMC6478623

[B58] Villa C, Combi R (2016) Potassium channels and human epileptic phenotypes: an updated overview. Front Cell Neurosci 10:81. 10.3389/fncel.2016.00081 27064559 PMC4811893

[B59] Zhao J, et al. (2022) Activated astrocytes attenuate neocortical seizures in rodent models through driving Na+-K+-ATPase. Nat Commun 13:7136. 10.1038/s41467-022-34662-2 36414629 PMC9681834

